# Spider‐Silk‐Inspired Tough, Self‐Healing, and Melt‐Spinnable Ionogels

**DOI:** 10.1002/advs.202305697

**Published:** 2023-11-23

**Authors:** Lijie Sun, Hongfei Huang, Luzhi Zhang, Rasoul Esmaeely Neisiany, Xiaopeng Ma, Hui Tan, Zhengwei You

**Affiliations:** ^1^ Center for Child Care and Mental Health (CCCMH) Shenzhen Children's Hospital Shenzhen 518038 China; ^2^ State Key Laboratory for Modification of Chemical Fibers and Polymer Materials, College of Materials Science and Engineering, Institute of Functional Materials, Research Base of Textile Materials for Flexible Electronics and Biomedical Applications (China Textile Engineering Society), Shanghai Engineering Research Center of Nano‐Biomaterials and Regenerative Medicine Donghua University Shanghai 201620 China; ^3^ Department of Materials and Polymer Engineering, Faculty of Engineering Hakim Sabzevari University Sabzevar 9617976487 Iran; ^4^ Biotechnology Centre Silesian University of Technology Krzywoustego 8 Gliwice 44‐100 Poland

**Keywords:** 3D printing, fibers, ionogels, self‐healing ionogels, sensors

## Abstract

As stretchable conductive materials, ionogels have gained increasing attention. However, it still remains crucial to integrate multiple functions including mechanically robust, room temperature self‐healing capacity, facile processing, and recyclability into an ionogel‐based device with high potential for applications such as soft robots, electronic skins, and wearable electronics. Herein, inspired by the structure of spider silk, a multilevel hydrogen bonding strategy to effectively produce multi‐functional ionogels is proposed with a combination of the desirable properties. The ionogels are synthesized based on *N*‐isopropylacrylamide (NIPAM), *N, N*‐dimethylacrylamide (DMA), and ionic liquids (ILs) 1‐ethyl‐3‐methylimidazolium bis(trifluoromethylsulfonyl)imide ([EMI][TFSI]). The synergistic hydrogen bonding interactions between PNIPAM chains, PDMA chains, and ILs endow the ionogels with improved mechanical strength along with fast self‐healing ability at ambient conditions. Furthermore, the synthesized ionogels show great capability for the continuous fabrication of the ionogel‐based fibers using the melt‐spinning process. The ionogel fibers exhibit spider‐silk‐like features with hysteresis behavior, indicating their excellent energy dissipation performance. Moreover, an interwoven network of ionogel fibers with strain and thermal sensing performance can accurately sense the location of objects. In addition, the ionogels show great recyclability and processability into different shapes using 3D printing. This work provides a new strategy to design superior ionogels for diverse applications.

## Graphical abstract

Inspired by the structure of spider silk, a multilevel hydrogen bonding strategy is proposed to effectively produce multi‐functional ionogels with a combination of mechanically robust, room‐temperature self‐healing capacity, facile processing, and recyclable properties. The ionogels have shown great potential for use in wearable electronics, electronic skins, and soft robotics. The molecular strategy will be powerful tools to develop high‐performance materials.

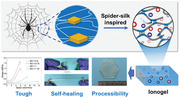

## 1 Introduction

Ionogel is a 3D network of a polymer matrix containing ionic liquids (ILs).^[^ ^1^ ^]^ Owing to the fascinating physicochemical properties of ILs including high ionic conductivity, thermal and (electro)chemical stability, low volatility, and nonflammability, ionogel possesses unique features, such as ionic conductivity, environmental stability, and flexibility.^[^ ^2^, ^3^, ^4^ ^]^ Consequently, ionogel has been an ideal candidate for use as ionic conductors, which has attracted a great deal of attention in a wide range of applications,^[^ ^1^, ^5^, ^6^, ^7^, ^8^, ^9^, ^10^, ^11^, ^12^, ^13^ ^]^ including wearable electronics, biomedical devices, and energy storage systems.

However, ionogel‐based devices are susceptible to damage and losing their original function during the service life, leading to the performance of the device attenuation, and even failure.^[^ ^14^, ^15^ ^]^ Inspired by living organisms, spontaneous self‐healing without any external stimuli at room temperature has developed and attracted substantial attention in the development of highly durable and sustainable materials.^[^ ^16^, ^17^ ^]^ To induce the self‐healing capacity and prolong service life, reversible non‐covalent interactions or dynamic covalent bonds are introduced into the ionogels. The high dynamic non‐covalent interactions (i.e., hydrogen bonds, ion‐dipole interactions, ion clusters, and metal‐ligand interactions) endow ionogels with room temperature self‐healing capability,^[^ ^16^, ^18^, ^19^, ^20^, ^21^, ^22^, ^23^ ^]^ while the most developed self‐healing ionogels suffer from low mechanical strength and it limits their further applications.^[^ ^2^, ^19^ ^]^ By contrast, the self‐healing ionogels based on dynamic covalent bonds (i.e., diselenide bonds, oxime‐urethane bonds, etc.) exhibited higher mechanical strength.^[^ ^15^, ^24^, ^25^, ^26^, ^27^ ^]^ However, dynamic covalent bonds always need more energy to achieve the dissociation/recombination or exchange, thus, the self‐healing of ionogels exhibits a lower efficiency and relies on external stimuli (heating or lighting). Despite significant progress in self‐healing ionogel, achieving simultaneous high mechanical robustness and self‐healing ability at ambient conditions, without any external stimuli, is still a formidable challenge.

In nature, spider silk is a typical high‐performance natural material, which displays a specific combination of properties having high strength, large extension, and high damping capacity.^[^ ^28^ ^]^ Theoretical and molecular dynamics simulation results ascribed the excellent mechanical properties of spider silk to the unique structure which comprises hard β‐sheet nanocrystals embedded in a soft semi‐amorphous protein matrix.^[^ ^29^, ^30^ ^]^ The hydrogen bonds are widely distributed in two‐phases of spider silk. Among them, the β‐sheet nanocrystals with dense hydrogen bonds play a decisive role in the tensile strength of spider silk. The hydrogen bonds in soft semi‐amorphous protein matrix are initially broken during the stretching process to dissipate a considerable amount of energy, resulting in the large extensibility of spider silk. The synergy between β‐sheet nanocrystals and a semi‐amorphous protein matrix imparts remarkable strength and toughness to spider silk. Recently, most high‐performance artificial materials have been developed based on this natural structure.^[^ ^31^, ^32^, ^33^, ^34^ ^]^ Poly(*N*‐isopropylacrylamide) (PNIPAM), a typical temperature‐responsive polymer, possesses an amphipathic nature with hydrophilic amide bonds (─CO─NH─) and hydrophobic isopropyl group (─CH(CH_3_)_2_). PNIPAM is a well‐known polymer with low critical solution temperature (LCST) in water and exhibits phase transition at a certain temperature.^[^ ^35^ ^]^ Owing to the reversible phase transition, PNIPAM‐based hydrogels have attracted widespread attention and found diverse promising applications such as smart coating, drug delivery, and artificial muscles.^[^ ^36^, ^37^, ^38^ ^]^ On the contrary, it is noteworthy that PNIPAM exhibits upper critical solution temperature (UCST) behavior in some ILs, e.g., 1‐ethyl‐3‐methylimidazolium bis(trifluoromethylsulfonyl)imide ([EMI][TFSI]).^[^ ^39^, ^40^ ^]^ At room temperature, hydrogen bonding interactions between amide bonds in PNIPAM are significantly stronger than those between the amide bonds of PNIPAM and [EMI][TFSI], leading to the aggregation of PNIPAM chains in [EMI][TFSI], which can simulate hard β‐sheet nanocrystals in spider silks.

Herein, inspired by the structure of spider silk, we proposed a multilevel hydrogen bonding strategy to effectively regulate ionogel properties such as high mechanical robustness and room temperature self‐healing capacity. Accordingly, the ionogels were developed based on PNIPAM chains, poly(*N*,*N*‐dimethylacrylamide) (PDMA) chains, and ILs ([EMI][TFSI]) to mimic the two‐phase structure of spider silk. The aggregation of PNIPAM chains with dense hydrogen bonding interaction strongly resembled the hard β‐sheet nanocrystals in spider silk. The PDMA and PNIPAM chains provided secondary hydrogen bonding interactions to simulate soft amorphous protein matrix in spider silk. The hydrogen bonding interaction between PDMA chains and [EMI][TFSI] increased the compatibility and effectively prevented leakage of [EMI][TFSI] from ionogel. The developed ionogel consequently showed superior mechanical strength and toughness by the energy dissipation effect from sacrificial physical interactions (hydrogen bonding) like spider silk. The hydrogen bonding interaction between PDMA chains and [EMI][TFSI] plasticized the polymer chains and promoted the mobility of polymer chains, which caused the fast polymer chain diffusion across the interface. With the dynamic and reversible nature of the hydrogen bonds of polymeric chains, the ionogel exhibited high‐efficiency self‐healing performance. Moreover, the hydrogen bonding interactions between PNIPAM chains would transform into hydrogen‐bonding interactions between PNIPAM chains and [EMI][TFSI] with the increasing temperature, the viscosity dropped rapidly and the ionogel could be easily processed. Correspondingly, the viscosity increased and the shape of ionogel was fixed with the temperature dropping. Therefore, the developed ionogel showed facile processing such as fused deposition modeling 3D printing, and spinning. Furthermore, due to their reversible nature, the ionogels could be recycled and repeatedly printed, a highly desired feature to reduce electronic waste.

## 2 Results and Discussion

### 2.1 Preparation and Characterization of the Ionogels

**Figure 1:**
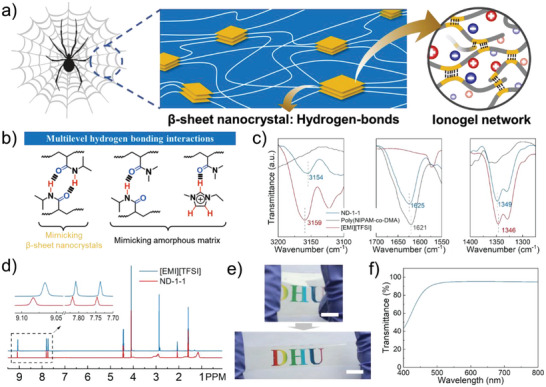
Design and characterization of the ionogel. a) Schematic spider‐silk structure with β‐sheet nanocrystals and spider‐silk‐inspired ionogel structure. b) Schematic illustration of the multilevel hydrogen bond interactions in ionogels. c) FTIR spectra of [EMI][TFSI], poly(NIPAM‐co‐DMA), and ionogel ND‐1‐1. d) ^1^H NMR spectra of [EMI][TFSI] and ionogel‐ND‐1‐1. e) Photographs of the ionogel ND‐1‐1 (scale bar: 1 cm). f) The transmittance spectrum of a 1.5  mm thickness ionogel film.

Spider silk with a two‐phase structure is composed of β‐sheet nanocrystals with dense hydrogen bonds and an amorphous organic biological matrix, which endows spider silk with high toughness (**Figure** **1**a). Inspired by the aforementioned structure, the PNIPAM and PDMA chains were introduced in ionogel structure to simulate β‐sheet nanocrystals and amorphous organic biological matrix and construct the tough ionogel. Herein, the ionogels were synthesized through an easy and cost‐efficient one‐pot photo‐initiated copolymerization of NIPAM and DMA in [EMI][TFSI] in the absence of any cross‐linkers (Figure S1, Supporting Information). As shown in Table S1 (Supporting Information), the molar ratio of NIPAM and DMA was systematically changed, to regulate the mechanical properties and self‐healing capacities of the ionogels. They are denoted as ND‐x‐y, where x‐y is the molar ratio of NIPAM and DMA, respectively, and the polymer without the ILs is denoted as poly(NIPAM‐co‐DMA). The ionogel networks are constructed by multilevel hydrogen bonds between polymer chains, as well as between ILs and polymer chains (Figure 1b). The aggregation of PNIPAM chains with dense hydrogen bonding interaction serves as a strong physical cross‐linking structure. By contrast, PDMA exhibits excellent compatibility with [EMI][TFSI] and can form hydrogen bonds with them.^[^ ^41^ ^]^ The hydrogen bond interactions of polymer chains and ILs in ionogels were confirmed using the proton nuclear magnetic resonance (^1^H NMR) spectra, Fourier transform infrared (FT‐IR), and differential scanning calorimetry (DSC). The ^1^H NMR spectra showed the peaks of H atoms of the imidazolium cation in ILs shift after the addition of polymer (Figure 1d), confirming the formation of hydrogen bonding between ILs and polymer chains.^[^ ^42^ ^]^ The same results were also obtained from the FT‐IR evaluations (Figure S2, Supporting Information). The absorbance bands located at 3159 cm^−1^ that corresponds to the stretching vibrations of C─H from the imidazole ring in ILs shifted to lower wavenumbers in ionogels (3154 cm^−1^), while the stretching vibrations of C═O at 1621 cm^−1^ in PDMA shifted to higher wavenumbers (1625 cm^−1^) in ionogels (Figure 1c).^[^ ^2^ ^]^ In addition, the asymmetric stretching vibrations of C─F from the [TFSI] anion in ILs shifted from 1346 to 1349 cm^−1^ in ionogels. The shifts indicate the presence of hydrogen bonds between poly(NIPAM‐co‐DMA) chains and ILs. The high miscibility of the ILs with the polymer led to the formation of an ionogel composite with high transparency (Figure 1e). The average transmittance of a 1.5 mm thickness film was determined to be more than 90% under visible‐light wavelengths of 500–800 nm, as shown in Figure 1f. Furthermore, the DSC tests were performed to investigate polymer dynamics (Figure S3, Supporting Information). The glass transition temperature (*T*_g_) of the ionogel increased from 6.5 to 13.5°C upon the increase of the NIPAM weight, indicating that the hydrogen bond interactions between PNIPAM chains limited the mobility of polymer chains in ionogels. Rheological tests on ionogel ND‐1‐1 showed the major role of the hydrogen bonding interactions in providing the physical cross‐linking structures in ionogels (Figure S4, Supporting Information). Owing to the strong physical cross‐linking structure between PNIPAM chains, the as‐prepared ionogel ND‐1‐1 showed good stretchability along with retained high transparency during stretching.

### 2.2 Mechanical Properties

**Figure 2:**
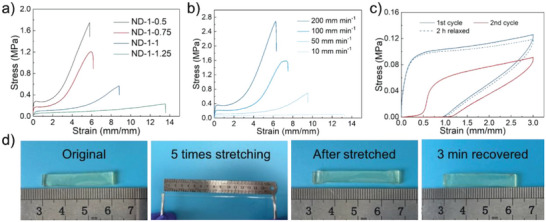
The mechanical properties of the ionogel. a) The typical stress–strain curves of the synthesized ionogels with different ratios of NIPAM and DMA. b) Tensile stress−strain curves of the ionogel ND‐1‐1 under several deformation rates. c) Repeated cyclic tensile curves of ionogel ND‐1‐1 at 300% strain. The first and second cycles of the tensile tests were carried out without any waiting time, while before the 3^rd^ cycle, the sample was allowed to relax for 120 min at 25 °C. d) Photographs of elastic resilience of the stretched ionogel ND‐1‐1 after 500% strain.

Uniaxial along with cyclic tensile tests were carried out to assess the mechanical properties of the ionogel with different monomer molar ratios of NIPAM and DMA since the mechanical properties of the ionogels are adjustable. As presented in **Figure** **2**a, with increasing the molar ratio of NIPAM, the tensile strength of ionogel noticeably increased from 0.23 ± 0.05 to 1.75 ± 0.23 MPa, and Young's modulus increased from 0.57 ± 0.22 to 4.41 ± 0.77 MPa. It was attributed to the increased amount of PNIPAM, which elevates the hydrogen bond cross‐linking density within the ionogel network. In contrast, the tensile strain of the ionogel decreased from 1375 ± 216% to 579 ± 87%, indicating the PNIPAM chains have a significant impact on the mechanical properties of ionogel. To further investigate the hydrogen bonding interaction for mechanical properties of ionogel, the ionogel ND‐1‐1 was selected. The mechanical properties of the ionogel‐ND‐1‐1 were found to be strongly dependent on the deformation rates of the test (Figure 2b). The Young's modulus and tensile strength of the ionogel ND‐1‐1 significantly increased with increasing strain rate. Increasing the crosshead speed from 10 to 200 mm min^−1^ caused a nearly 29‐fold and 13‐fold increase in tensile strength and Young's modulus, respectively, while the tensile strain decreased from ≈1300% to ≈600%. Through the rheological test in the frequency sweep mode of ionogels, it was shown that the storage modulus (G’) and loss modulus (G’’) values increased with the increase of frequency, and the G’’ was higher than G’ at low frequency, while lower at high frequency, confirming the hydrogen bonding cross‐linked structure (Figure S4, Supporting Information). Moreover, with increasing the molar ratio of NIPAM, G’ increased significantly, and the crossover point of G’ and G’’ shifted to lower frequencies, indicating the increase of hydrogen bonding cross‐linking density. Therefore, it was indicated that the dense hydrogen bonding interaction of the aggregation in the PNIPAM chains was dynamic in ionogel.^[^ ^43^ ^]^ Owing to the multilevel hydrogen bonding interaction in ionogel, the weaker hydrogen bonds could serve as sacrificial bonds to endow ionogel with high toughness (Figure S5, Supporting Information). To demonstrate it, repeated cyclic tensile tests of ionogel ND‐1‐1 at a 300% strain, considered a large strain, were carried out (Figure 2c). The cyclic tests were performed back‐to‐back for two cycles without any waiting time. The large hysteresis loop in the first cycle confirmed a notable energy dissipation, while in the second cycle, it was significantly less than that acquired in the first cycle. It can be attributed to the limited time frame prevented the broken hydrogen bonds, as sacrificial bonds, from returning to their initial state. For the 3rd cyclic the specimen was allowed to relax for 120 min at 25 °C before the tensile test. After the relaxation, the sample showed a loading–unloading curve like that of the first cycle, confirming good toughness and elasticity recovery of ionogel ND‐1‐1. The elasticity of ionogel would be more visual by the photographs of the elastic resilience of the stretchable ionogel after 500% strain. It can be seen that the ionogels are highly stretchable (more than 500%) (Figure 2d), then, with the removed force, the ionogel immediately recovered (Figure 2d). After 30 min, the ionogel returned to full length.

### 2.3 Self‐Healing Performance

**Figure 3:**
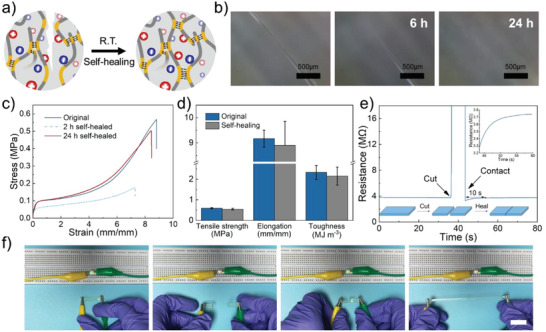
Self‐healing performance of ionogels. a) Schematic illustration of the healing process of ionogel. b) Optical microscopy images of ionogel ND‐1‐1 film after being scratched with a perfect self‐healing performance at room temperature. c) Tensile stress–strain curves of original and healed ionogel ND‐1‐1. d) Tensile strength, elongation at break, and toughness of original and healed ionogel ND‐1‐1. e) Resistance versus test time during the cutting‐healing process for the ionogel ND‐1‐1. Insert: a zoomed‐in view of the resistance changes. f) Demonstration of the stretchable and self‐healing properties of ionogel ND‐1‐1 in series with a LED (scale bar: 2 cm).

As a highly desirable feature for future materials, the self‐healing capacity can significantly prolong the material lifetime to ensure the device's reliable, stable operation in the long term.^[^ ^44^ ^]^ However, achieving both high mechanical properties and healing efficiency in mild conditions, especially at ambient temperature, remains a major challenge for self‐healing materials.^[^ ^19^, ^45^, ^46^ ^]^ Owing to multilevel dynamic hydrogen bonding interactions, the tough ionogel showed excellent self‐healing performance, without any external stimuli, at room temperature (**Figure** **3**a). The self‐healing behaviors of ionogel were investigated in detail. Therefore, the surface scratch recovery test was first carried out on ionogel ND‐1‐1 using a 30–50 µm wide blade to scratch the film. Optical microscopy images showed almost complete self‐healing within 24 h at ambient conditions, as presented in Figure 3b. Furthermore, the bulk and quantitively self‐healing assessments for the synthesized ionogel were also conducted. The strip was divided into two halves, and then the separated parts were brought close to each other for 24 h at room temperature. The healing efficiency of ionogels was determined from *η* = *P*_H_/*P*_0_, where *P*_H_ was the value of tensile strength, elongation, or toughness of healed ionogels, *P*_0_ was the value of tensile strength, elongation, or toughness of original ionogels. After healing, the tensile stress‐strain curves of healed ionogel ND‐1‐1 and ionogel ND‐1‐1.25 were similar to those obtained for the pristine ionogels (Figure 3c; Figure S6, Supporting Information). Particularly, the tensile strength, elongation, and toughness of pristine ionogel ND‐1‐1 were determined to be 0.59 ± 0.03 MPa, 916 ± 34% and 2.33 ± 0.34 MJ m^−3^, respectively, while those obtained for the healed ionogel ND‐1‐1 were 0.51 ± 0.04 MPa, 890 ± 95% and 2.02 ± 0.46 MJ m^−3^, respectively (Figure 3c,d). The healing efficiency in tensile strength, elongation, and toughness of ionogel ND‐1‐1 was more than 90%. Interestingly, with increasing the molar ratio of NIPAM, the elongation at break of healed ionogel ND‐1‐0.5 and ionogel ND‐1‐0.75 could recover 97% and 98% of their pristine values, respectively, while the tensile strength only recovered 71% and 76% after healing for 24 h at room temperature (Figure S6, Supporting Information). The influence of ILs content and temperature on the self‐healing properties of ionogels was investigated. When the ionic liquid was absent, poly(NIPAM‐co‐DMA) was incapable of self‐healing after 24 h at room temperature (Figure S7, Supporting Information). With the introduction of ILs, the plasticizing effect increased the mobility of polymeric chains to promote the self‐healing properties of the ionogel (Figure S8, Supporting Information). The high temperature was able to disrupt hydrogen bonding within the ionogel system and accelerate chains mobility, thereby enabling rapid self‐healing. The ionogel ND‐1‐1 could fully heal within 30 min at 80 °C (Figure S9, Supporting Information). Furthermore, the real‐time changes in resistance during the cutting‐healing process of the ionogel ND‐1‐1 were monitored using a digital multimeter to investigate the self‐healing capacity of the ionogels in electrical conductivity (Figure 3e). The healing efficiency of electrical conductivity was determined from *η* = 1‐(*R*_H_−*R*_0_)/*R*_0_, where *R*_H_ was the resistances of the ionogel ND‐1‐1 after healed, and *R*_0_ was the original resistances. When the ionogel was cut apart, the conductive pathway failed, and the resistance of the material increased rapidly to infinity. Once the two separated halves came into close contact, the conductive pathway rapidly recovered in 40 ms, and the resistance of the ionogel ND‐1‐1 recovered to 99% of its initial value after 10 s. Figure 3f and Movie S1 (Supporting Information) indicate this performance more visually. The ionogel strip as an ionic conductor can be connected to the circuit with a green light‐emitting diode (LED). When the ionogel was cut into two pieces, the LED was off. While the two pieces of ionogel ND‐1‐1 came into contact, the LED was lighted immediately. The two cut pieces healed without any external stimuli in 5 min. Furthermore, the healed ionogel could be stretched to 300%. The mechanism of the self‐healing performances of ionogel can be proposed as follows. During the self‐healing process, ILs could serve as a plasticizer to effectively increase the mobility of the polymer chains to the healing interface,^[^ ^26^, ^47^ ^]^ the weaker hydrogen bonds were recombined rapidly, then the stronger hydrogen bonds between PNIPAM chains were reformed slowly. Thus, the electrical conductivity of ionogel was instantaneously recovered, followed by elongation recovery. Subsequently, tough hydrogen bonds between PNIPAM chains which played a leading role in the strength of ionogel further recombined along the mobility of polymer chains. Hence, the strength of ionogel needed more time to heal, while the partial strength was difficult to recover with increasing the molar ratio of NIPAM. Noticeably, the LED in the circuit became less bright with an increase in strain deformation, indicating that the resistance change of ionogel ND‐1‐1 was sensitive to deformation. This implied that ionogels had the potential in the application of strain sensors. Therefore, the synthesized ionogel with excellent self‐healing performance could be employed with more durability and less cost of maintenance and replacement.

### 2.4 Processibility of Ionogel

**Figure 4:**
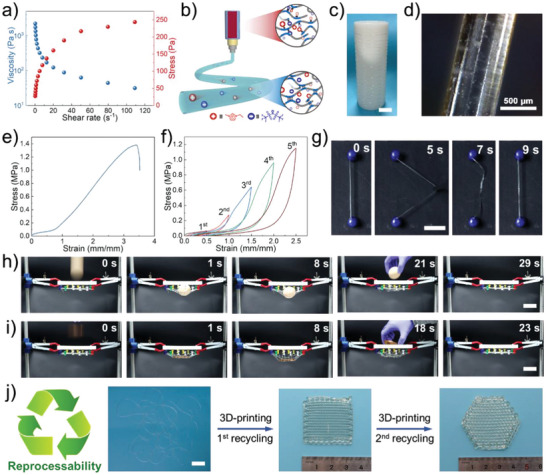
Processibility of ionogel. a) Viscometry results of the ionogel ND‐1‐1 (stress and viscosity versus shear rates) at 145 °C. b) Schematic representation of the melt extrusion process and chemical structure of ionogel. c) Photograph of a long single fiber collected on a continuously winding drum spool (scale bar: 2 cm). d) Optical microscope photograph of ionogel fiber. e) Stress‐strain curve of ionogel fiber. f) Stress‐strain curve of ionogel fiber with a step‐cycle loading. g) Photograph of an ionogel fiber showing the hysteresis effect during its recovery process (scale bar: 2 cm). The ionogel fiber net caught free‐falling h) egg and i) copper billet (scale bar: 4 cm). j) The reprocessability of ionogel fiber (scale bar: 4 cm).

Owing to the dynamics of hydrogen bonds, the hydrogen bonds initially formed between PNIPAM chains gradually shift towards forming hydrogen bonds between PNIPAM chains and [EMI][TFSI] as the temperature increases. It results in a reduction of the strong hydrogen bond cross‐linking density within the ionogels. Consequently, the viscosity decreases significantly, making the ionogel easily processable. To investigate the processibility of ionogel, the rheological test of ionogel ND‐1‐1 was performed. The temperature sweep experiment of ionogel ND‐1‐1 showed that the G’ and G’’ values decreased with the increase in temperature, revealing the weaker hydrogen bonding cross‐linked structure at high temperatures (Figure S10, Supporting Information). For the temperature higher than 145 °C, the G’ and G’’ of the ionogel markedly decreased. In order to obtain a better processing performance of ionogel ND‐1‐1, the processing temperature was selected as 145 °C which was far less than the thermal decomposition temperature (Figure S11, Supporting Information). Furthermore, the viscosity and stress of ionogel ND‐1‐1 at different shear rates and constant temperature of 145 °C were evaluated and shown in **Figure** **4a**. The viscosity was 2200 Pa s at a low shear rate (0.1 s^−1^), while it dropped to ≈31 Pa s at a high shear rate (100 s^−1^)., which is perfect for the extrusion process. Here, ionogel fibers were fabricated through a continuous melt‐spinning process. At high temperatures, the disentanglement of the physically cross‐linked network led to a significant decrease in the viscosity, as a shear‐thinning behavior, which is desirable for melt‐spinning to re‐construct ionogel architectures (Figure 4b). After cooling, the recombination of hydrogen bonds and rearrangements of polymeric chains form physically cross‐linked networks of ionogels, which can realize continuous melt spinning to obtain the ionogel fibers. A single ionogel fiber was successfully fabricated by melt‐spinning and collected (Figure 4c).

The ionogel fibers showed a cylindrical shape with a consistent diameter (700–800 µm) evaluated by optical microscopy (Figure 4d). A thinner fiber with a ca. 500 µm diameter could be prepared by increasing the winding speed (Figure S12, Supporting Information). Furthermore, both uniaxial and cyclic tensile tests were conducted to assess the mechanical properties of the ionogel fiber. The tensile strength and strain at break of ionogel fiber were determined to be 1.42 ± 0.32 MPa and 355 ± 39%, respectively (Figure 4e). Compared to bulk ionogel, the ionogel ND‐1‐1 fiber exhibited higher tensile strength and lower strain. It could be attributed to the rearrangement of hydrogen bonds during high‐temperature processing, resulting in the formation of larger‐sized hydrogen‐bond interactions and an orderly arrangement of the structure during the extrusion process. The ionogel fiber with multilevel hydrogen bonding interaction exhibited spider‐silk‐like features with strain softening and hysteresis, which raised with the increase in the strain (Figure 4f), indicating that the breakage of weak hydrogen bonds could dissipate a large amount of energy.^[^ ^48^ ^]^ Furthermore, cyclic tensile test results revealed that the mechanical properties of ionogel fiber could return to the original state after a relaxation period of 1 h (Figure S13, Supporting Information). Upon stretching to 200% strain, the dissipated energy was 0.54 MJ m^−3^. Spider silk shows hysteresis behavior upon deformation and recover.^[^ ^49^ ^]^ The spider‐silk‐like hysteresis behavior of ionogel fiber is more obvious in Figure 4g and Movie S2 (Supporting Information). When the straight and tight ionogel fiber was subjected to stretching and subsequently released, it initially became loose and gradually returned to its original length within 4 s.

To further demonstrate the energy dissipation effect of ionogel fibers, we weaved them into a net to be used for catching falling objects (Figure S14, Supporting Information). A 43 g egg and 100 g copper billet were selected as test models and freely fell from a height of 20 cm (Figure 4h,i). It was observed that the objects almost no re‐bounced after contact with the ionogel fiber net (Movie S3, Supporting Information). Subsequently, the ionogel fiber net underwent gradual deformation. When the egg and copper billet were removed, the net rapidly returns to its original state. It indicated that the ionogel fibers possessed spider‐silk‐like features with excellent energy dissipation performance.

The ionogel‐ND‐1‐1 exhibited excellent processability. It could not only be obtained as 1D ionogel fibers but also as 2D direct‐writing ionogel patterns and 3D objects with complicated structures through the 3D printing process (Movie S4; Figures S15‐S17, Supporting Information). Owing to the dynamic of the physical cross‐linking structure based on hydrogen bonds, the processed ionogel fibers could be recycled and reprocessed to grid‐structured ionogel film with high stretchability (Figure 4j; Figure S18, Supporting Information). Moreover, the recycled ionogel fiber were subjected to mechanical characterization. Compared to original ionogel ND‐1‐1 fiber, the tensile strength of the recycled fibers recovered 85%. It could be attributed to the degradation of the polymer network caused by the high‐temperature processing.

In contrast to the reported representative room temperature self‐healing ionogels in recent years (Table S2, Supporting Information), the ionogels based on multilevel hydrogen bonds showed outstanding comprehensive properties including, stress, strain, self‐healing, processibility and recyclability.

### 2.5 Sensing Performance

**Figure 5:**
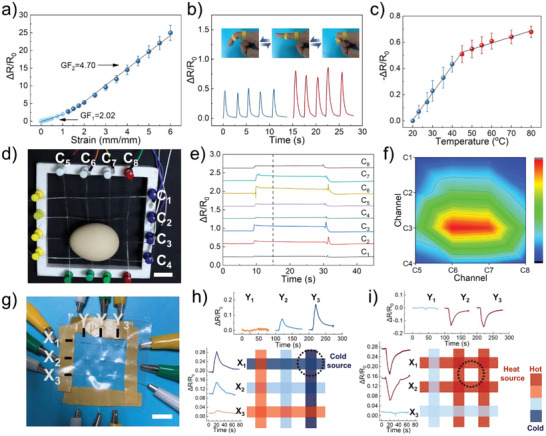
Sensing performance of ionogel ND‐1‐1. a) Relationship between changes in the relative resistance of ionogel ND‐1‐1 sensors and strain. b) Relative resistance changes of the ionogel ND‐1‐1 under different index finger angles. c) The change in the resistance of ionogel ND‐1‐1 with temperature. d) Photograph of the interlacing fiber sensor array of 4 × 4 strain‐sensitive elements (scale bar: 2 cm). e) The strain detection of the fiber‐based sensor array. f) The signal of fiber strain‐sensor array for sensing position of the egg. g) Photograph of the interlacing fiber sensor array of 3 × 3 temperature‐sensitive elements (scale bar: 2 cm). The ability of the fiber sensor array to detect the changes in ambient temperature, as well as locate the h) cold or i) heat source.

ILs provide a high ionic conductivity to the ionogel. Considering the ionic conductivity and mechanical properties, the ionogel has promising potential to be employed as strain sensors. In the current research, the performance of the ionogel ND‐1‐1 as strain and temperature sensors was investigated. The relationship between the relative resistance of an ionogel‐based sensor and its strain is presented in **Figure** **5a**. The relative resistance of the ionogel‐based sensor increased with an increase in the strain from 0 to 600%, demonstrating that the ionogel had a broad sensing range. The gauge factor (GF), defined as the ratio of the relative resistance change to the applied strain, is an important parameter for the quantitative analysis of the sensitivity of a strain sensor. The ionogel‐based sensor had a GF of 2.02 for the strain lower than 100% and a GF of 4.7 for strains higher than 100%, which is superior to the several reported ionogel‐based strain sensors.^[^ ^15^, ^26^, ^47^, ^50^ ^]^ As presented in Figure 5b, the ionogel ND‐1‐1 had a perfect ability to accommodate the stretching movement of the finger according to its mechanical adaptability. The resistance of the ionogel ND‐1‐1 rose as soon as the finger bent, because of elongation of ionogel during the finger bent. In addition, the finger angle could be identified and differentiated by measuring the amount of the resistance change. The resistance of ionogel ND‐1‐1 was repeatable variation with strain during dynamic process. Figure S20 (Supporting Information) shows the ionic conductivity of ionogels is temperature dependent. The increasing temperature resulted in an increase in ionic conductivity due to the ions move more easily at high temperatures, which agrees with the movement of ions in the polymer matrix. Thus, the ionogel was evaluated to demonstrate its temperature‐sensing ability. As shown in Figure 5c, the temperature‐sensing performance of ionogel ND‐1‐1 included two different linear regions.

Fiber‐based electronic devices having integration features including flexibility, breathability, and comfort have drawn remarkable attention in the field of wearable electronics.^[^ ^5^, ^51^, ^52^, ^53^, ^54^, ^55^, ^56^ ^]^ Similar to bulk ionogel, the ionogel fibers possessed strain and temperature‐sensing capabilities.^[^ ^57^ ^]^ Furthermore, the ionogel fiber could be rationally woven to construct a sensor array with high sensing accuracy. Consequently, a 4 × 4 strain‐sensitive sensor array based on ionogel fibers was fabricated (Figure 5d). The ionogel fibers in the sensor array were named C_1_ to C_8_. When an egg was placed in a sensor array, the resistance of ionogel fiber immediately underwent a corresponding change because of the change in the strain (Figure 5e). The precise position of the egg could be determined by accurately measuring the varying degree of resistance change in each fiber (Figure 5f; Figure S21, Supporting Information). Moreover, the sensor array could determine the object's precise position using the temperature variation of ionogel fibers. To evaluate the capability of the sensors for object mapping, a 3 × 3 sensor array was constructed (Figure 5g). Such a sensor array is employed to detect the presence of heat or cold sources. For demonstration, the heat source (a glass containing water) with temperatures of 10 and 40 °C was close to/away from the sensor array (Figure S22, Supporting Information) and then checked the response behaviors of each fiber (name as X_1_, X_2_, X_3_, Y_1_, Y_2_, and Y_3_) in the sensor array. The results confirmed that the fiber sensor had ability to respond to the variations in the temperature (Figure 5h). The resistance of fibers X_1_, X_2_, Y_2_, and Y_3_ decreased simultaneously when the 40 °C heat source was close to the sensor array (Figure 5i), while fibers X_3_ and Y_1_ were far away from the heat source without the change of resistance. As the heat source moved away, the resistance immediately increased. When the fiber sensor was near to a 10 °C cold source, the resistance change was reversed to fiber sensor for 50 °C. Through the resistance change process of fibers in the sensor array, the position and length of time of the heat source can be accurately confirmed. Thus, the developed sensor array with precise strain and temperature detecting ability presented a potential application in intelligent perception.

## 3 Conclusion

In summary, inspired by the structure of spider silk, a multilevel hydrogen bonding strategy is proposed to achieve high mechanical robustness and simultaneously maintain the autonomous healing ability of ionogels at room temperature. The ionogels integrated characteristics of transparency, high stretchability, conductivity, self‐healing capacity, facile processibility, and recyclability, which are all highly desirable properties for next‐generation electronics. Importantly, for the first time, ionogel fibers could be continuously produced via facile melt spinning. In combination with the 3D printing process, the ionogel fibers can construct diverse electronic devices with complicated and heterogeneous 2D and 3D structures. The interwoven networks of ionogel fiber are similar to cobwebs that can dissipate external loading energy and accurately sense the location of objects based on strain and thermal sensing performance. Furthermore, the ionogels can be recycled and repeatedly printed, which is a highly desired feature to reduce electronic waste. The ionogels have shown great potential for use in wearable electronics, electronic skins, and soft robotics. This design principle of multilevel hydrogen bonds will pave the path toward the development of high‐performance materials.

## 4 Experimental Section

### Materials

NIPAM and 2‐Hydroxy‐2‐methylpro‐piophenone (initiator 1173) were purchased from TCI Co., Ltd. DMA was provided by J&K Chemical Co. Ltd. 1‐ethyl‐3‐methylimidazolium bis(trifluoromethylsulfonyl) imide ([EMI][TFSI]), ≥99% was provided by the Lanzhou Institute of Chemical Physics, CAS. Deuterated acetone was obtained from Cambridge Isotope Laboratories, Inc. All the other solvents were purchased from Sinopharm Chemical Reagent. All the reagents were used as received without further purification.

### Preparation of the Ionogels

The ionogel was synthesized through a one‐pot strategy. NIPAM and DMA were mixed in a certain weight ratio, and subsequently, [EMI][TFSI] (55 wt.% relative to polymer monomer) was added to the mixture. Then, the initiator 1173 (0.2 wt.% relative to polymer monomer) which could initiate free radical polymerization of NIPAM and DMA was added to the mixture. The resulted mixture was then heated to 60 °C and magnetically stirred for 3 min to obtain a homogeneous solution. After bubbling nitrogen, the solution was degassed in a vacuum chamber. Subsequently, the solution was poured into a quartz glass mold comprising a PDMS spacer sandwiched between two transparent glass plates. The resulted gels were cured using an UV light cross‐linker (UPP0404A, Uvata (Shanghai) Precision Optoelectronics Co., Ltd.) for 30 min at a wavelength of 365 nm. Finally, place the ionogel in a plastic dish for 24 h.

### 3D Printing of the Ionogel

The synthesized ionogels were printed utilizing a commercially pressure‐controlled direct ink 3D printer (BS4.2, GESIM). The ionogels were first loaded into an extrusion cartridge. After melting at 145 °C for 10 min, the homogeneous, low‐viscosity, and transparent ionogels were extruded utilizing a 0.7 mm diameter flat tip needle at an extrusion speed of 6 mm s^−1^.

### Characterizations and Measurements

^1^H NMR spectra were recorded on a Bruker AVANCE 600 NMR spectrometer. A Thermo Scientific Nicolet 8700 spectrometer was employed to record the attenuated total reflectance FTIR (ATR‐FTIR) spectra of the synthesized samples. The optical transmittance of the prepared ionogel films was evaluated using a Jasco V‐630 UV–vis spectrophotometer. The mechanical properties of the prepared ionogels were evaluated by an MTS E42 tensile machine equipped with a 100 N sensor, while the uniaxial tensile tests were conducted at a cross‐head rate of 50 mm min^−1^, unless otherwise noted. Impedance spectroscopy was recorded utilizing a CHI670E electrochemical analyzer. Thermogravimetric analysis (TGA) tests were conducted on a TG 209 F1 thermogravimetric analyzer (NETZSCH, Germany) in the temperature range of 40–600 °C under N_2_ atmosphere and a heating rate of 10 °C min^−1^. Moreover, DSC tests were carried out utilizing a DSC‐822 (Mettler Toledo, Switzerland) at a heating rate of 10 °C min^−1^ under N_2_ atmosphere. The electrical resistance was recorded on a Keithley DMM7510 multimeter. Additionally, the relative resistance change (∆*R*/*R*_0_) was determined from (∆*R*/*R*_0_) = (*R*−*R*_0_)/*R*_0_, where *R*_0_ and R represent the resistances of the ionogel before and after stretching or pressing, respectively. The rheological tests were carried out using a TA Instruments Discovery HR‐2 rheometer and TA Instruments ARES‐G2 rheometer using a 25 mm plate–plate geometry.

## Conflict of Interest

The authors declare no conflict of interest.

## Supporting information

Supporting Information

Supplemental Movie1

Supplemental Movie2

Supplemental Movie3

Supplemental Movie4

## Data Availability

The data that support the findings of this study are available from the corresponding author upon reasonable request.
