# Supplementary material for: Spider‐Silk‐Inspired Tough, Self‐Healing, and Melt‐Spinnable Ionogels
Source: Adv Sci (Weinh). 2023 Nov 23;11(3):2305697. doi: 10.1002/advs.202305697 (PMC10797445; doi:10.1002/advs.202305697)
Supplement: Supplementary file 1 — Supporting Information [file ADVS-11-2305697-s002.pdf]

ADVANCED SCIENCE Open Access

Supporting Information

for *Adv. Sci.*, DOI 10.1002/advs.202305697

Spider-Silk-Inspired Tough, Self-Healing, and Melt-Spinnable Ionogels

Lijie Sun, Hongfei Huang, Luzhi Zhang, Rasoul Esmaeely Neisiany, Xiaopeng Ma, Hui Tan* and Zhengwei You**

Supporting information for

Spider-silk-inspired tough, self-healing, and melt-spinnable ionogels

Lijie Sun[1,2], Hongfei Huang[2], Luzhi Zhang[1,2], Rasoul Esmaeely Neisiany[3,4], Xiaopeng Ma[1], Hui Tan[1]*, Zhengwei You[2]**

[1]Center for Child Care and Mental Health (CCCMH), Shenzhen Children's Hospital, 518038 Shenzhen, China.

[2]State Key Laboratory for Modification of Chemical Fibers and Polymer Materials, College of Materials Science and Engineering, Institute of Functional Materials, Research Base of Textile Materials for Flexible Electronics and Biomedical Applications (China Textile Engineering Society), Shanghai Engineering Research Center of Nano-Biomaterials and Regenerative Medicine, Donghua University, 201620 Shanghai, China.

[3]Department of Materials and Polymer Engineering, Faculty of Engineering, Hakim Sabzevari University, Sabzevar, 9617976487, Iran.

[4]Biotechnology Centre, Silesian University of Technology, Krzywoustego 8, 44-100, Gliwice, Poland.

*Corresponding Author. E-mail: maxiaopeng@126.com (X.M.); huitan@email.szu.edu.cn (H.T.); zyou@dhu.edu.cn (Z.Y.)

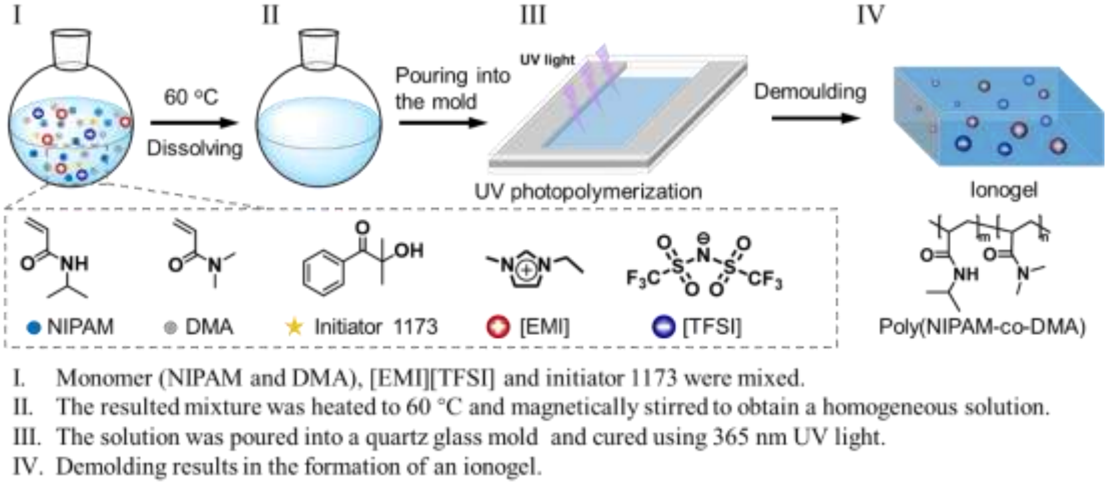

Figure S1. Schematic illustration of the preparation of the ionogels through one-pot synthesis.

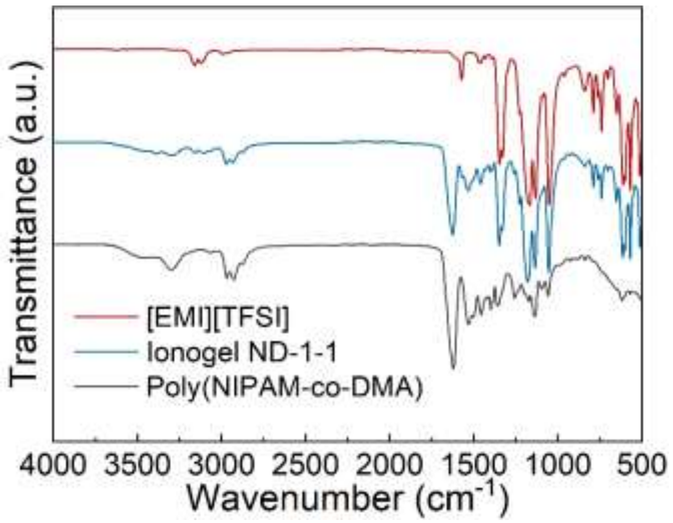

Figure S2. FTIR spectra of [EMI][TFSI], poly(NIPAM-co-DMA), and ionogel ND-1-1 from 4000 to 500 cm^{-1}.

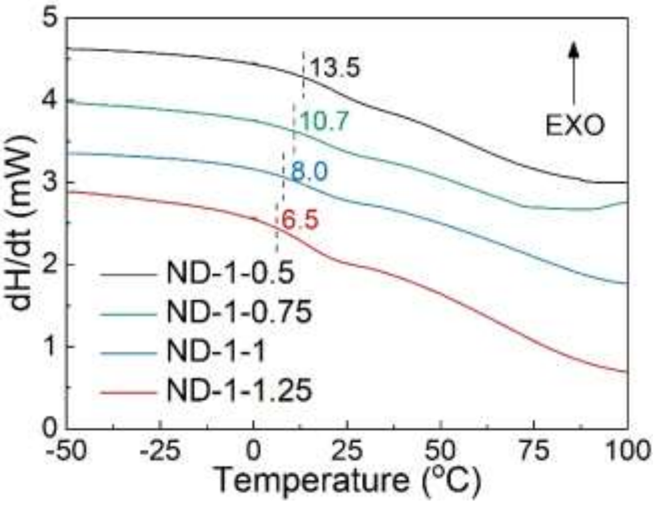

Figure S3. DSC curves of the ionogels.

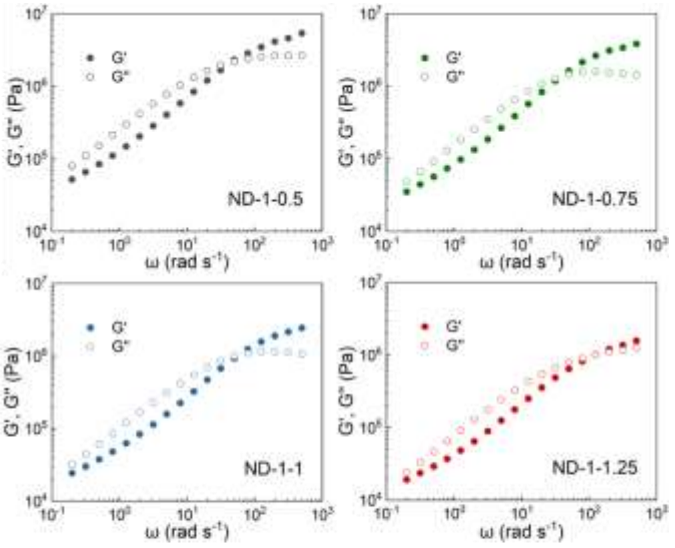

Figure S4. Rheological tests: Frequency dependence of storage (G′) and loss (G″) moduli for the ionogels at 30 °C.

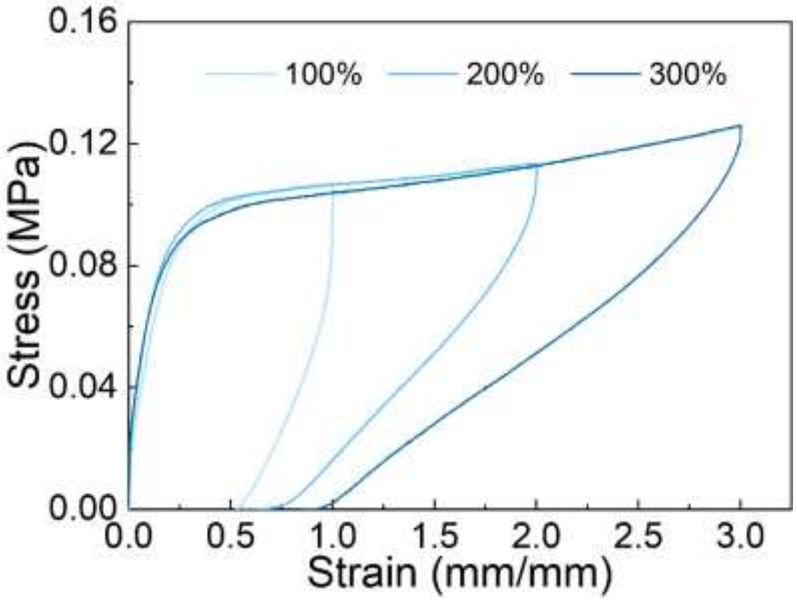

Figure S5. The stress-strain curves of ionogel ND-1-1 during a cyclic stretching/releasing process at different strains (100, 200 and 300%)

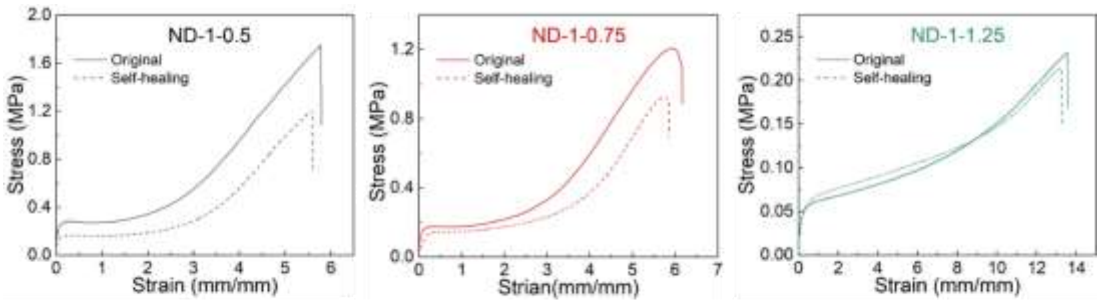

Figure S6. Tensile stress–strain curves of original and healed ionogels ND-1-0.5, ND-1-0.75 and ND-1-1.25.

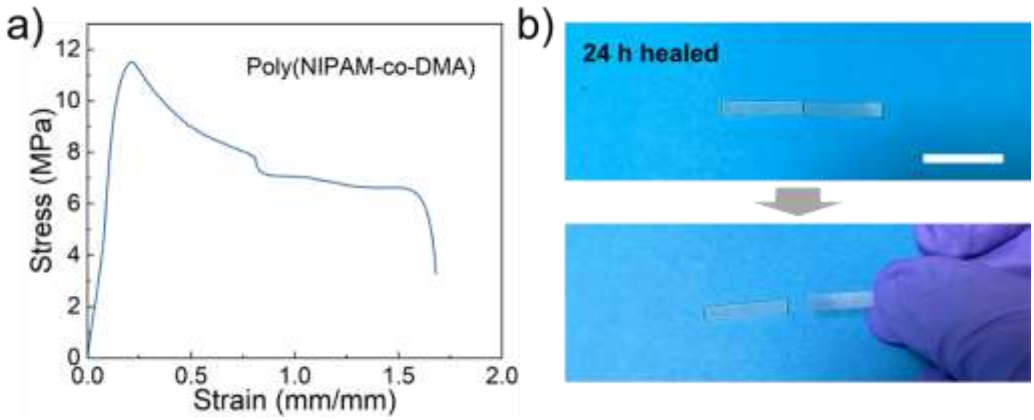

Figure S7. a) Tensile stress–strain curves of poly(NIPAM-co-DMA). b) The photograph of poly(NIPAM-co-DMA) after 24 h at room temperature. Poly(NIPAM-co-DMA) was incapable of

self-healing.

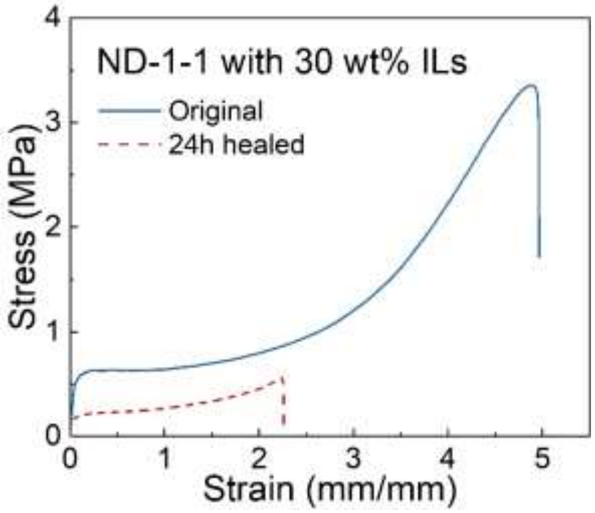

Figure S8. Tensile stress–strain curves of original and healed ionogel ND-1-1 with 30 wt% ILs at room temperature.

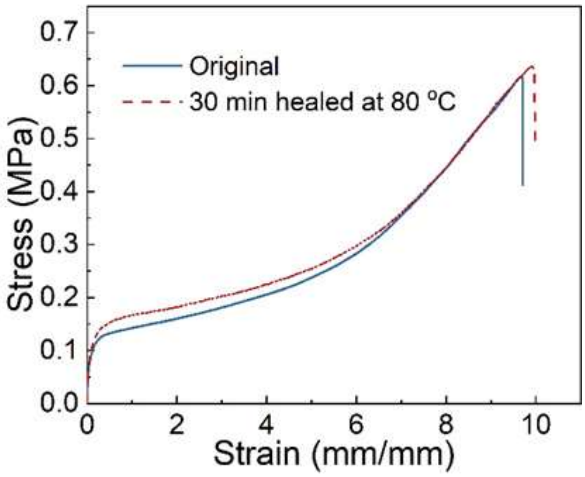

Figure S9. Tensile stress–strain curves of original and healed ionogel ND-1-1 at 80 ^{o}C.

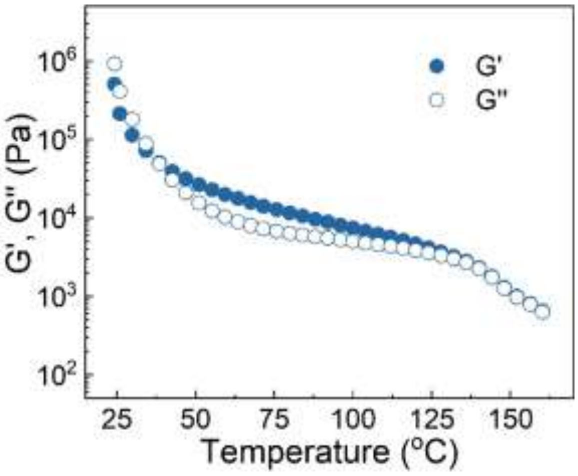

Figure S10. Changes of G′ and G″ with increasing temperature from 25 to 160 °C for the ionogel ND-1-1.

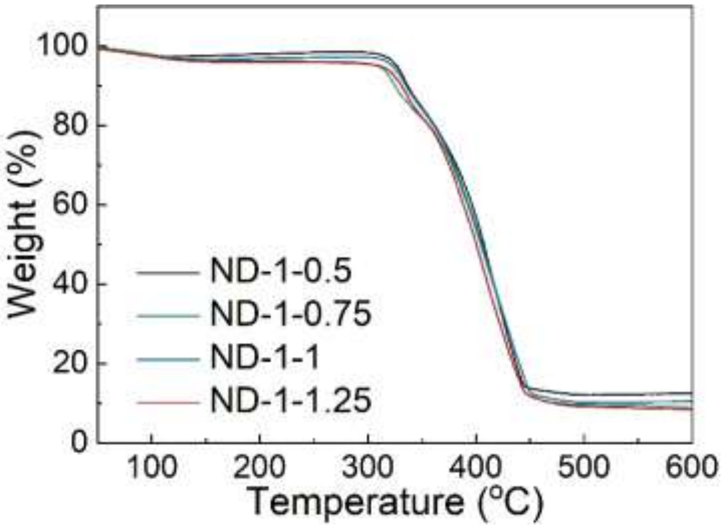

Figure S11. TGA curves of the ionogels.

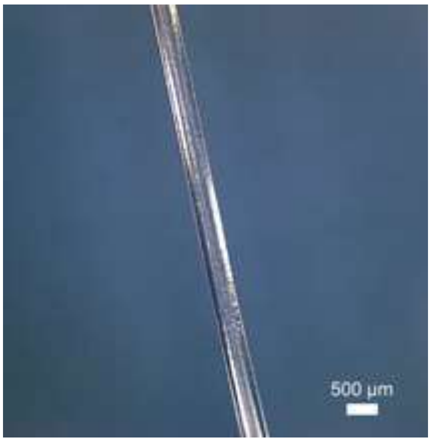

Figure S12. Optical microscope photograph of ionogel fiber

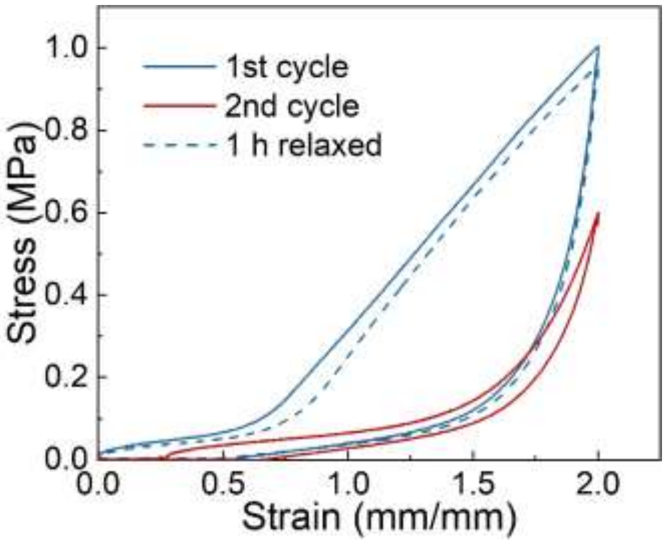

Figure S13. Repeated cyclic tensile curves of ionogel fiber at 200% strain. There was no waiting time between two consecutive cyclic tensile (cycle 1 and cycle 2). The sample was then allowed to relax for 1 h before the 3rd cyclic tensile test.

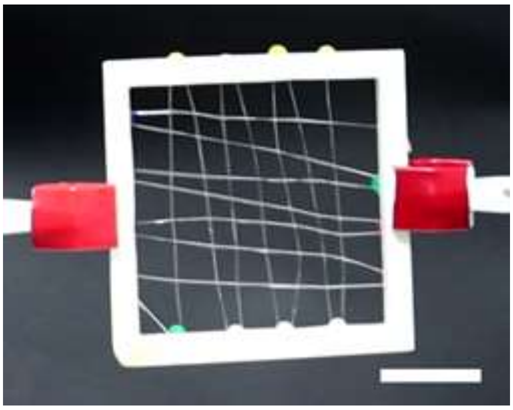

Figure S14. A net woven from ionogel fibers (scale bar: 4 cm).

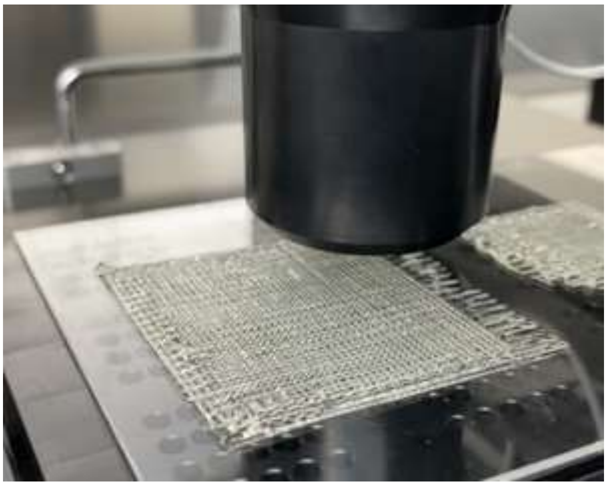

Figure S15. The photo of 3D printing process of ionogel ND-1-1.

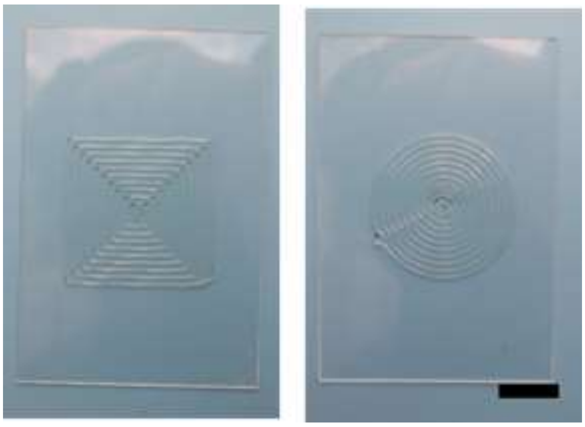

Figure S16. The photograph of direct writing patterns of ionogel ND-1-1 by 3D printing (scale bar: 2 cm).

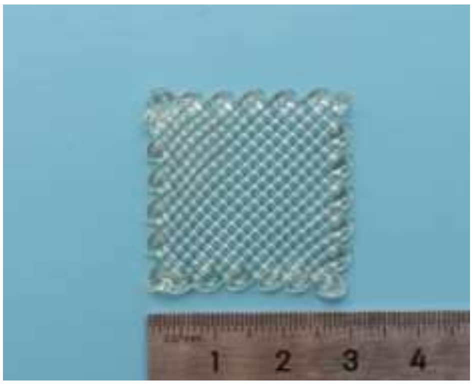

Figure S17. The photograph of 3D printing ionogel with grid structures.

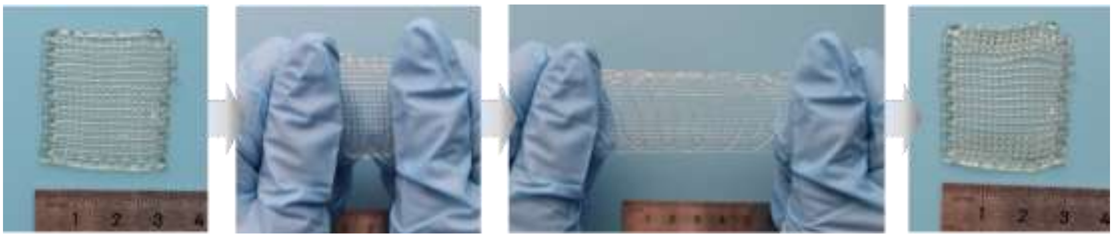

Figure S18. The photo of the stretchablity and recovery of 3D printing ionogel ND-1-1.

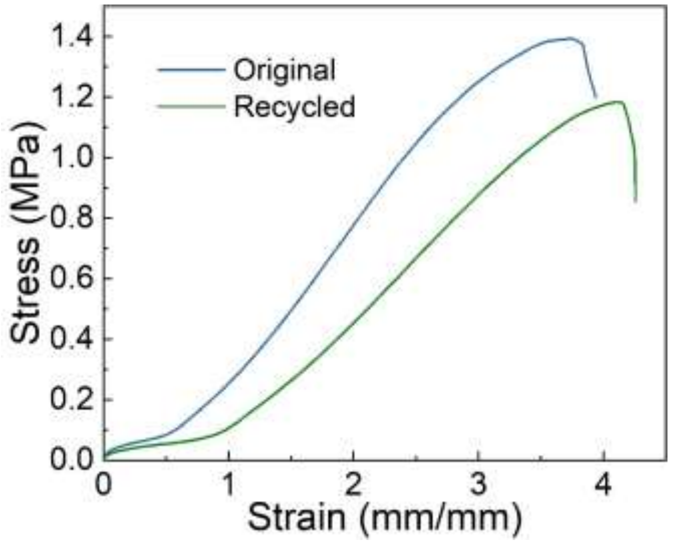

Figure 19. Stress–strain curves of the original and recycled ionogel ND-1-1 fibers.

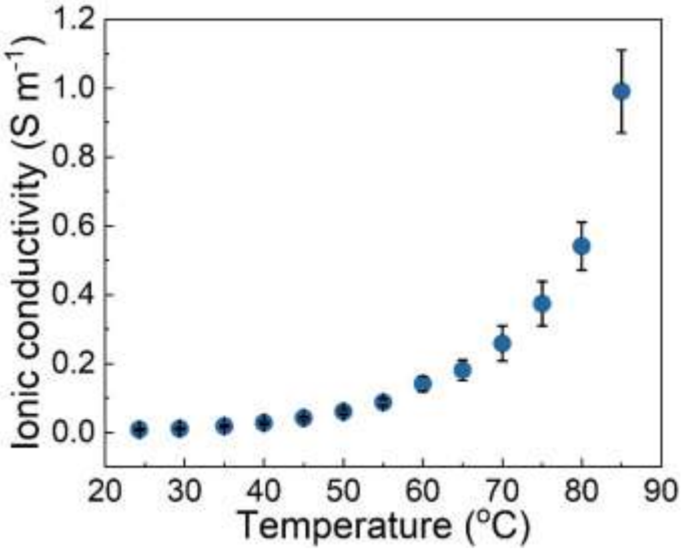

Figure S20. Influence of temperature on the ionic conductivity of the ionogel ND-1-1.

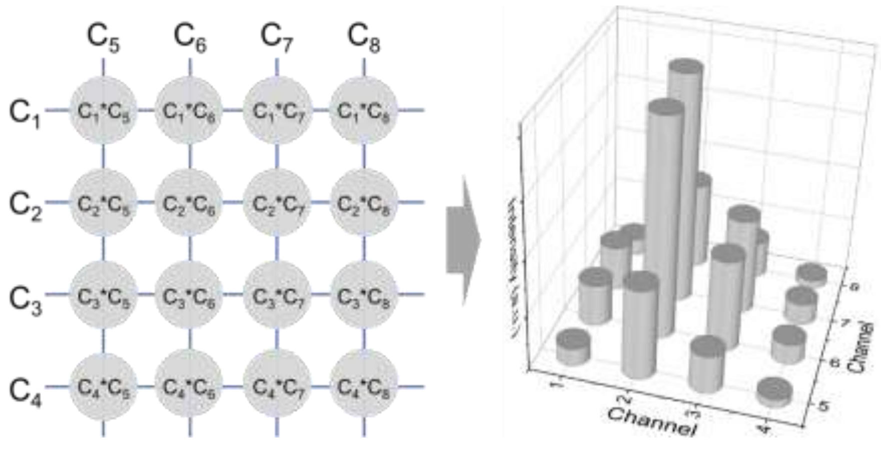

Figure S21. The signal processing of fiber strain-sensor array for sensing position of the egg at

15s in Figure 5e. Sixteen signals were obtained through the cumulative in resistance change values

Samples	[EMI][TFSI] *wt%* (relative to polymer monomer)	Monomer molar ratio (NIPAM/DMA)	Initiator 1173 *wt%* (relative to polymer monomer)
ND-1-0.5	55	100:50	0.2
ND-1-0.75	55	100:75	0.2
ND-1-1	55	100:100	0.2
ND-1-1.25	55	100:125	0.2

of each fiber at the crossover points.

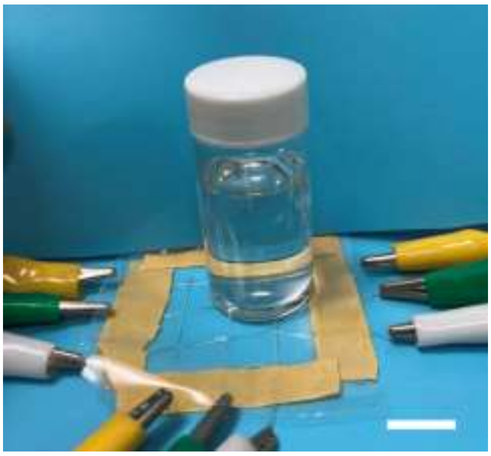

Figure S22. A glass holding water with different temperature (40 °C) as heat source was close to temperature sensor array (scale bar: 2cm).

Table S1. Composition of the ionogels.

Table S2. The performance comparison between the prepared ionogels and the representative ionogels that have been reported in recent years

Crosslinker	Tensile strength (MPa)	Strain (%)	Healing efficency at room temperature (%)	Process-ibility	Recycl-ability	Ref.
imine bonds	1.66	400	95	-	-	[1]
Disulfide bonds	0.3	500	99 (UV irradiation)	3D printing	-	[2]
Hydrogen bond	0.32	400	98	-	-	[3]
Hydrogen bond, ionic interactions	0.27	3200	98	-	Yes	[4]

Hydrogen bond	0.55	1800	99	-	-	[5]
Hydrogen bond, dipole interactions	0.22	500	50	-	-	[6]
Coordinatio n bond	0.05	1000	98	-	-	[7]
Metal-ligand interactions	0.58	30	98	-	-	[8]
Hydrogen bond	0.2~1.75	1300~500	71~97	3D printing	Yes	This work

[1] Y. Fu, L. Chen, F. Xu, X. Li, Y. Li, J. Sun, *J. Mater. Chem. A* **2022**, 10, 4695.

[2] M. Zhang, X. Tao, R. Yu, Y. He, X. Li, X. Chen, W. Huang, *J. Mater. Chem. A* **2022**, 10, 12005.

[3] R. Tamate, K. Hashimoto, T. Horii, M. Hirasawa, X. Li, M. Shibayama, M. Watanabe, *Adv. Mater.* **2018**, 30, 1802792.

[4] W. Li, L. Li, S. Zheng, Z. Liu, X. Zou, Z. Sun, J. Guo, F. Yan, *Adv. Mater.* **2022**, 34, 2203049.

[5] L. Xu, Z. Huang, Z. Deng, Z. Du, T. L. Sun, Z. H. Guo, K. Yue, *Adv. Mater.* **2021**, 33, 2105306.

[6] L. Sun, H. Huang, Q. Ding, Y. Guo, W. Sun, Z. Wu, M. Qin, Q. Guan, Z. You, *Adv. Fiber Mater.* **2022**, 4, 98.

[7] W. Liao, X. Liu, Y. Li, X. Xu, J. Jiang, S. Lu, D. Bao, Z. Wen, X. Sun, *Nano Res.* **2022**, 15, 2060.

[8] J. Wu, L. Huang, S. Wang, X. Li, L. Wen, X. Li, T. Feng, P. Li, Z. Fang, M. Wu, W. Lv, *Energy Storage Mater.* **2023**, 57, 549.
